# Modulation of long noncoding RNA (lncRNA) and messenger RNA (mRNA) expression in the liver of Beagle dogs by *Toxocara canis* infection

**DOI:** 10.1186/s13071-023-05738-9

**Published:** 2023-03-29

**Authors:** Yang Zou, Wen-Bin Zheng, Hany M. Elsheikha, Jun-Jun He, Yi-Xin Lu, Shuai Wang, Aijiang Guo, Xing-Quan Zhu

**Affiliations:** 1 State Key Laboratory for Animal Disease Control and Prevention, Key Laboratory of Veterinary Parasitology of Gansu Province, Lanzhou Veterinary Research Institute, Chinese Academy of Agricultural Sciences, Lanzhou, Gansu Province 730046 People’s Republic of China; 2 Laboratory of Parasitic Diseases, College of Veterinary Medicine, Shanxi Agricultural University, Taigu, 030801 Shanxi Province People’s Republic of China; 3 Faculty of Medicine and Health Sciences, School of Veterinary Medicine and Science, University of Nottingham, Loughborough, LE12 5RD UK; 4 Key Laboratory of Veterinary Public Health of Higher Education of Yunnan Province, College of Veterinary Medicine, Yunnan Agricultural University, Kunming, Yunnan Province 650201 People’s Republic of China; 5 Heilongjiang Key Laboratory for Zoonosis, College of Veterinary Medicine, Northeast Agricultural University, Harbin, 150030 Heilongjiang Province People’s Republic of China

**Keywords:** *Toxocara canis*, RNA-seq, Long non-coding RNAs, Co-location, Liver, Beagle dogs

## Abstract

**Background:**

Long non-coding RNAs (lncRNAs) and messenger RNAs (mRNAs) play crucial roles in regulating various physiological and pathological processes. However, the role of lncRNAs and mRNAs in mediating the liver response during *Toxocara canis* infection remains incompletely understood.

**Methods:**

In the present study, the expression profile of lncRNAs and mRNAs was investigated in the liver of Beagle dogs infected by *T. canis* using high-throughput RNA sequencing.

**Results:**

Compared with the control groups, 876 differentially expressed (DE) lncRNAs and 288 DEmRNAs were identified at 12 h post-infection (hpi), 906 DElncRNAs and 261 DEmRNAs were identified at 24 hpi, and 876 DElncRNAs and 302 DEmRNAs were identified at 36 days post-infection (dpi). A total of 16 DEmRNAs (e.g. *dpp4*, *crp* and *gnas*) were commonly identified at the three infection stages. Enrichment and co-localization analyses identified several pathways involved in immune and inflammatory responses during *T. canis* infection. Some novel DElncRNAs, such as LNC_015756, LNC_011050 and LNC_011052, were also associated with immune and inflammatory responses. Also, LNC_005105 and LNC_005401 were associated with the secretion of anti-inflammatory cytokines, which may play a role in the healing of liver pathology at the late stage of infection.

**Conclusions:**

Our data provided new insight into the regulatory roles of lncRNAs and mRNAs in the pathogenesis of *T. canis* and improved our understanding of the contribution of lncRNAs and mRNAs to the immune and inflammatory response of the liver during *T. canis* infection.

**Graphical Abstract:**

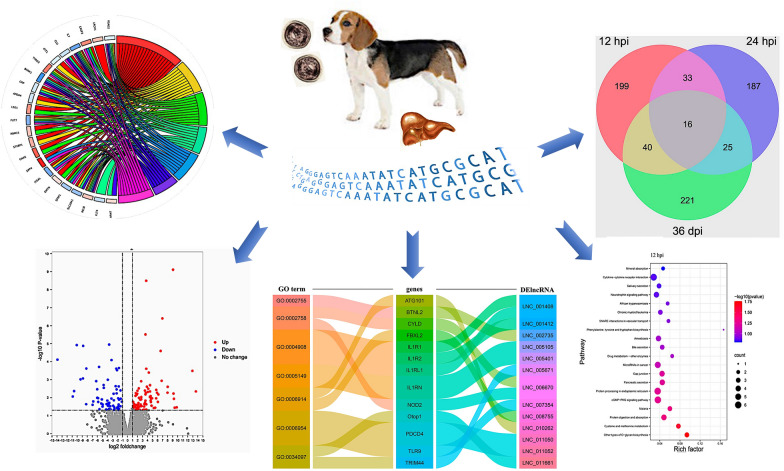

**Supplementary Information:**

The online version contains supplementary material available at 10.1186/s13071-023-05738-9.

## Background

*Toxocara canis*, a ubiquitous nematode in dogs, is responsible for the disease known as toxocariasis. Dogs infected by *T. canis* excrete eggs which contaminate the soil and the dog’s hair [1]. When *T. canis* eggs are ingested by the definitive canid host, the larvae hatch in the intestine, penetrate the intestinal wall and migrate to the mesenteric lymph nodes and reach the liver by 24 h post-infection (hpi). Subsequently, the larvae migrate to the lung at approximately 96 hpi following a cardio-pulmonary route. In the lungs, larvae break out into the alveoli and ascend through the trachea to the pharynx from where they are swallowed and enter the gastrointestinal tract to complete their development into adult roundworms [2].

*Toxocara canis* can also infect humans where the larvae migrate throughout the body without reaching the adult stage [3]. Clinical disease in humans can take several forms including visceral toxocariasis, ocular toxocariasis, neurotoxocariasis and covert toxocariasis [4]. Mice infected by *T. canis* exhibit pulmonary inflammation and increased levels of IgE, eosinophils and Th-2 type cytokines [5–7]. Migration of *T. canis* larvae through the livers can cause hepatic damage and inflammation [2]. A previous study showed the significant role of miRNAs in the pathogenesis of *T. canis* during the hepatic phase of parasite development in Beagle dogs [8].

The recent advances in understanding the molecular pathogenesis of *T. canis* infection in dogs has been facilitated by the application of high-throughput ‘omics’ approaches, including transcriptomics, proteomics and metabolomics [9]. For example, previous transcriptomic analysis allowed the identification of long noncoding RNA (lncRNA) and messenger RNA (mRNA) differentially expressed in the spleen [10] and lung [11] of Beagle dogs during *T. canis* infection. LncRNAs are cellular RNA transcripts with a length of > 200 nt [12] and regulate gene expression through a variety of mechanisms [13, 14]. They are involved in the regulation of diverse biological processes, including mediating host-parasite interaction [15–17].

Despite the advances in the understanding of the pathobiology of *T. canis* infection, the roles of lncRNAs in the hepatic immune-inflammatory response to infection are still incompletely defined. Therefore, we focused on the identification of lncRNAs and mRNAs involved in hepatic pathology associated with *T. canis* infection. In the present study, RNA-Seq analysis was performed to investigate the effect of *T. canis* infection on the expression of lncRNAs and mRNAs in the liver of Beagle dogs at different stages post-infection.

## Methods

### Ethics approval

The study was approved by the Research Ethics Committee of Lanzhou Veterinary Research Institute, Chinese Academy of Agricultural Sciences (approval no. 2018–015). All animals were under constant veterinary care during the study and were housed, fed and handled in strict compliance with accepted animal welfare guidelines.

### *Toxocara canis* infection

The *T. canis* eggs were isolated from the uteri of female worms and incubated at 28 °C and 85–95% relative humidity for 28 days. Then, the embryonated eggs were stored at 4 °C (in 1% formalin solution) as previously described [18]. The study protocol used to establish *T. canis* infection was described previously [18]. Briefly, 6–7-week-old Beagle puppies (*n* = 18) were allocated to six groups, with three dogs per group [8]. Three infected groups were inoculated orally with 1 ml saline containing 300 *T. canis* embryonated eggs/puppy, whereas dogs in the three control groups were inoculated with 1 ml saline only. All dogs were in apparent good health prior to and at the start of the study. Additionally, thorough health observations of each dog were performed at least daily during the study.

### Sample collection and RNA isolation

At 12 h post-infection (hpi), 24 hpi and 36 days post-infection (dpi), one infected group and one control group of puppies were euthanized by intracardiac injection of potassium chloride (KCl) solution under general anesthesia using Zoletil™ 50 (Virbac, Carros, France). This procedure was performed by a well-trained veterinarian with good knowledge of the anesthetic techniques to ensure the suitability of the anesthetic depth prior to administration of KCl. The livers were dissected from each puppy and washed with saline to remove the blood from the liver surface. Twenty-five milligram sample from the left lateral hepatic lobe was collected from each animal and stored in liquid nitrogen. No visible pathological changes were observed in the liver because of low infection of *T. canis* eggs. The total RNA was extracted from the liver sample using TRIZOL (Life Technologies, Carlsbad, CA, USA). The hepatic tissue samples were homogenized using liquid nitrogen, followed by lysis using 1 ml of TRIZOL reagent. The RNA was then extracted using chloroform, and the aqueous phase was obtained by centrifugation at 11,500 g for 15 min at 4 °C. The aqueous phase was transferred to a new RNA-free EP tube, and RNA was precipitated using isopropanol. Finally, the RNA samples were washed with 75% ethanol. Genomic DNA was removed from the RNA samples using DNaseI (New England BioLabs, Ipswich, MA, USA). Then 1% agarose gel was used to detect any degradation and contamination in the extracted RNA. Then, the RNA concentration and integrity were measured, and only RNA with integrity number (RIN) ≥ 8 was used in sequencing analysis [19].

### Library preparation and sequencing

Each lncRNA library was constructed with 3 μg rRNA-depleted RNA and was generated using NEBNext^®^ Ultra^™^ Directional RNA Library Prep Kit for Illumina® (NEB, USA). The first-strand cDNA was synthesized using random hexamer primer and M-MuLV Reverse Transcriptase (RNaseH-). To select cDNA fragments of ~ 150 to 200 bp length, the library fragments were subjected to purification using the AMPure XP system (Beckman Coulter, Beverly, MA, USA). Subsequently, 3 μl USER Enzyme (NEB, Ipswich, USA) was applied to the size-selected cDNA fragments, which were ligated with adaptors, and the mixture was incubated at 37 °C for 15 min. The mixture was then heated to 95 °C for 5 min and subjected to amplification. The PCR was carried out using Phusion High-Fidelity DNA polymerase, with Universal PCR primers and Index (X) Primer. The PCR products were purified using the AMPure XP system, and the library quality was evaluated on the Agilent Bioanalyzer 2100 system [20]. The index-coded samples were clustered using the TruSeq PE Cluster Kit v3-cBot-HS (Illumina) on a cBot Cluster Generation System, following the manufacturer's instructions. Subsequently, the generated clusters were sequenced on an Illumina Hiseq 4000 platform, producing 150-bp paired-end reads for the libraries.

### Identification of lncRNAs and mRNAs

The raw reads (raw data) of fastq format were obtained. Then, the adapter, poly-N and low-quality reads were removed to obtain the clean reads (clean data). The GC content was calculated to assess the quality of the data. The HISAT2 v2.0.4 was used to align clean reads to the *Canis lupus familiaris* reference genome [21]. StringTie (v1.3.1) was used to assemble the mapped reads [22]. Cuffdiff (v2.1.1) was used to calculate the FPKMs (fragments per kilobase of exon model per million fragments) of lncRNAs and mRNAs [23]. The splicing transcripts with exons ≥ 2 and lengths > 200 bp were screened for lncRNA transcripts. Three tools, CNCI (Coding-Non-Coding-Index) (v2), CPC (Coding Potential Calculator) (0.9-r2) and Pfam Scan (v1.3) (v20121028), were used to predict novel lncRNAs [24–26].

### Differential expression analysis

The differential expression analysis at each infection stage was performed using DESeq 2 (1.14.1) [27]. *P*-value and fold change were set as criteria for identifying the differential transcripts for lncRNAs and mRNAs. *P*-value < 0.05 and log2 (fold change) |≥ 1 were considered as differentially expressed (DE) lncRNA and DEmRNA.

### Functional analysis of DEmRNAs

Gene ontology (GO) annotation analysis of the DEmRNAs was performed using the GOseq R package [28], including biological process (BP), cellular component (CC) and molecular function (MF). Kyoto Encyclopedia of Genes and Genomes (KEGG) pathway enrichment analysis was used to map genes and identify the enriched signaling pathway using KOBAS 3.0 [29]. *P*-value < 0.05 was considered significant enrichment.

### Target gene prediction and GO analysis of DElncRNA

LncRNAs change the expression of nearby genes; thus, the potential target genes of DElncRNAs were predicted according to the co-location (within the upstream and downstream 100 kb) of mRNAs-DElncRNAs. GO annotation analysis was performed using the potential target genes of DElncRNA to predict the functions of DElncRNA. *P* < 0.05 was considered significant enrichment. The network between DElncRNAs and DEmRNAs was visualized using Cytoscape v.3.5 [30].

### Quantitative real-time PCR (qRT-PCR) analysis

Seven DEmRNAs and three DElncRNAs were randomly chosen to verify the RNA-Seq results using qRT-PCR as described previously [11]. Briefly, the PrimeScript™ RT reagent kit with gDNA Eraser (Takara, Tokyo, Japan) and lnRcute lncRNA cDNA first-chain synthesis kit (TianGen, Beijing, China) were used to synthesize the first-strand cDNA of the mRNA and lncRNA, respectively. Then, qRT-PCR was performed using a LightCycler480 (Roche, Basel, Switzerland). The amplification conditions for mRNAs were initial denaturation (95 °C for 10 min) followed by amplification and quantification program repeated for 40 cycles (95 °C for 30 s, 60 °C for 1 min, 60 °C for 1 min). The amplification condition of lncRNA included an initial denaturation (95 °C for 3 min) and 40 cycles of template denaturation at 94 °C for 5 s and annealing at 60 °C for 15 s.

The melting curve analysis was included in each reaction. The transcripts and primers are listed in Table 1. The ribosomal protein *L13A* was used as the housekeeping gene [11]. All reactions were repeated independently three times. The relative expression level was calculated by the 2^−ΔΔCt^ method [31].

**Table 1:** Transcripts and primers used in the qRT-PCR analysis

RNAs	Primer	Sequence (5ʹ to 3ʹ)	RNAs	Primer	Sequence (5ʹ to 3ʹ)
L13A-F (normalization control)	Reverse primer	GCCGGAAGGTTGTAGTCGT	L13A-R	Reverse primer	GGAGGAAGGCCAGGTAATTC
LNC_018761-F (lncRNA)	Forward primer	GACAGACACCGATTCCAGTATG	LNC_018761-R	Forward primer	AACACAGCTCTCTGGCTTTC
LNC_008052-F (lncRNA)	Reverse primer	GAGCAGCCTCACTGACAAA	LNC_008052-R	Reverse primer	GGGAACTCAGCACCATGAA
LNC_020932-F (lncRNA)	Forward primer	TTGGAAGAGTTAGGGCTTGAG	LNC_020932-R	Forward primer	CGGGAGGTCACCATATTGAT
ENSCAFT00000032207-F (mRNA)	Reverse primer	AGAGCACATGATTCGCTACC	ENSCAFT00000032207-R	Reverse primer	CCTTGTTTGTCCAATGCTTCTC
UGT2B31-F (mRNA)	Forward primer	CTCACCCACTCTTACCACATTT	UGT2B31-R	Forward primer	GAGCTCTGGACAAACTCTTCC
DLA88-F (mRNA)	Reverse primer	GTGTCCCTAGGAGCATAATGTG	DLA88-R	Reverse primer	CAGGAAAGCACATCAGAGAAGA
ENSCAFT00000047303-F (mRNA)	Forward primer	GTAAACGGCGGGAGTAACTATG	ENSCAFT00000047303-R	Forward primer	GACAGTGGGAATCTCGTTCATC
IGFBP1-F (mRNA)	Reverse primer	ACAGTTTCTACCTGCCCAAC	IGFBP1-R	Reverse primer	GTACACGCACCAGCAGAG
ENSCAFT00000043798-F (mRNA)	Forward primer	GTGTTCCTGGAGAACGTGAT	ENSCAFT00000043798-R	Forward primer	CGCTTGAGCGCGTAGAC
SAA1-F (mRNA)	Reverse primer	GGGAACTCAGCACCATGAA	SAA1-R	Reverse primer	GAGCAGCCTCACTGACAAA

## Results

### RNA-sequencing data

In this study, 2,119,950,482 raw reads were obtained from liver samples of 18 puppies. We identified 2,068,104,062 clean reads and 310.22 Gb clean data. The averages of GC and Q30 in the clean reads were 52.47% and 93.73%, respectively (Table 2). After mapping to the *C. lupus familiaris* reference genome, the transcripts were divided into different sub-types. A total of 23,928 mRNAs, 883 annotated lncRNAs and 20,694 novel lncRNAs transcripts were identified.

**Table 2:** Summary of the characteristics of lncRNA libraries

Groups	Samples	Raw reads	Clean reads	Bases (Gb)	Error rate (%)	Q30 (%)	GC content (%)
12 hpi	A12hC1	114542314	111829426	16.77	0.02	92.97	52.28
	A12hC2	110154550	105703084	15.86	0.02	93.04	51.48
	A12hC3	111062278	107848292	16.18	0.02	93.15	51.78
	A12hT1	126333980	122444414	18.37	0.02	92.33	52.6
	A12hT2	115618286	111971282	16.8	0.02	91.84	53.95
	A12hT3	126187564	122775802	18.42	0.02	91.95	51.68
24 hpi	B24hC1	121938966	119396404	17.91	0.02	94.5	53.3
	B24hC2	118084678	115069188	17.26	0.02	94.18	53.13
	B24hC3	121698022	119543532	17.93	0.02	94.63	52.57
	B24hT1	115306076	111615648	16.74	0.02	93.37	52.49
	B24hT2	113096264	110186886	16.53	0.02	94.26	52.69
	B24hT3	109523726	107116360	16.07	0.02	94.48	52.55
36 dpi	D36dC1	119474152	117429314	17.61	0.02	94.62	53.07
	D36dC2	123517866	121516894	18.23	0.02	94.49	52.53
	D36dC3	117239050	115115698	17.27	0.02	94.44	52.98
	D36dT1	112937468	110695554	16.6	0.02	94.3	51.55
	D36dT2	122780744	120148122	18.02	0.02	94.49	51.49
	D36dT3	120454498	117698162	17.65	0.02	94.06	52.29

### DElncRNAs and DEmRNAs

**Fig. 1:**
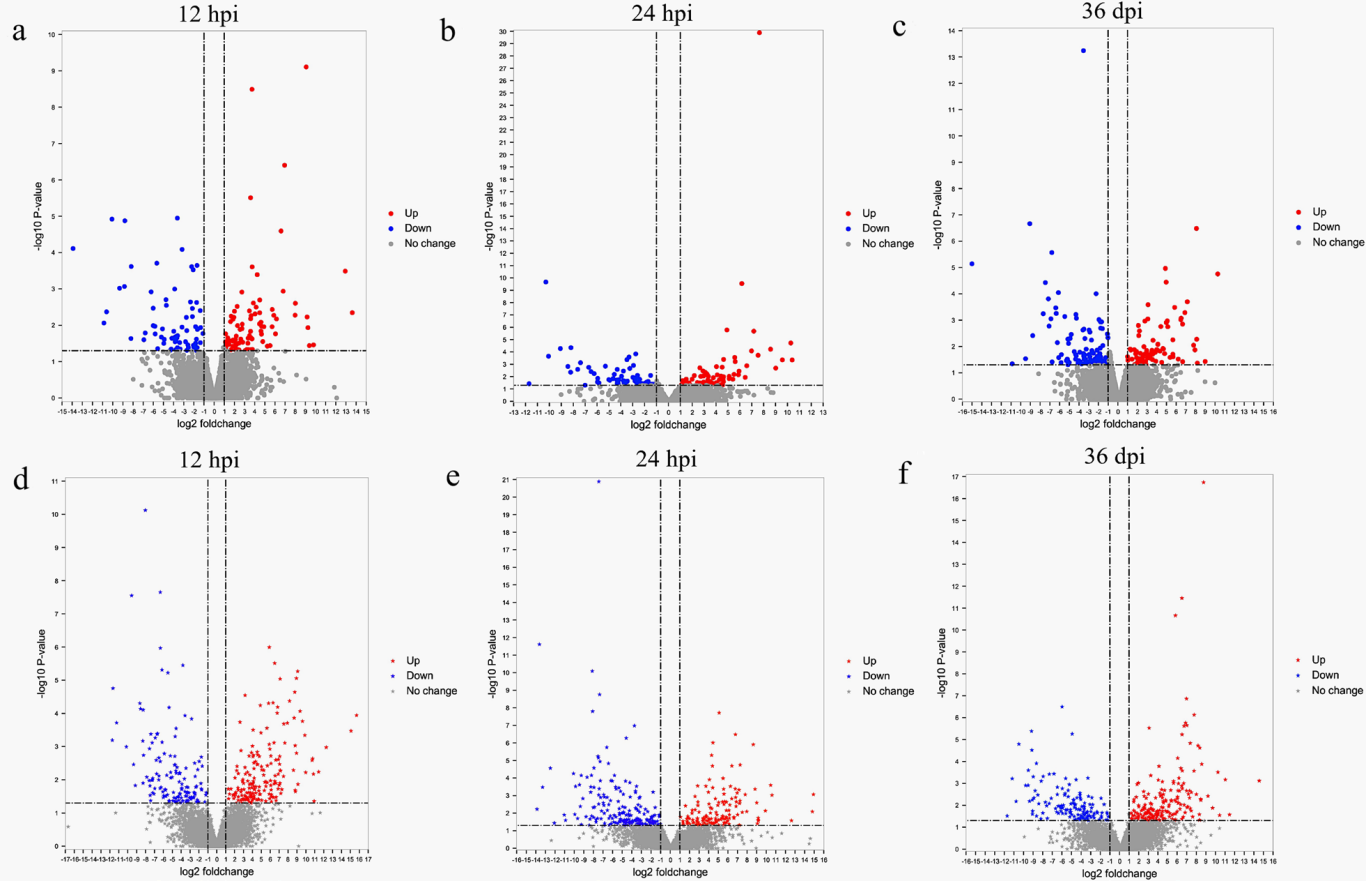
Volcano plots showing the differentially expressed (DE) mRNAs (**a**–**c**) and DElnRNAs (**d**–**f**) at 12 hpi, 24 hpi and 36 dpi, respectively. The X-axis shows the log2 fold change of the DERNAs, while the Y-axis shows the corresponding -log_10_ *P*-value. Up- and downregulated RNAs are shown in red and blue, respectively. DERNAs that did not pass the threshold for the log fold change (not significant) are shown in gray

**Fig. 2:**
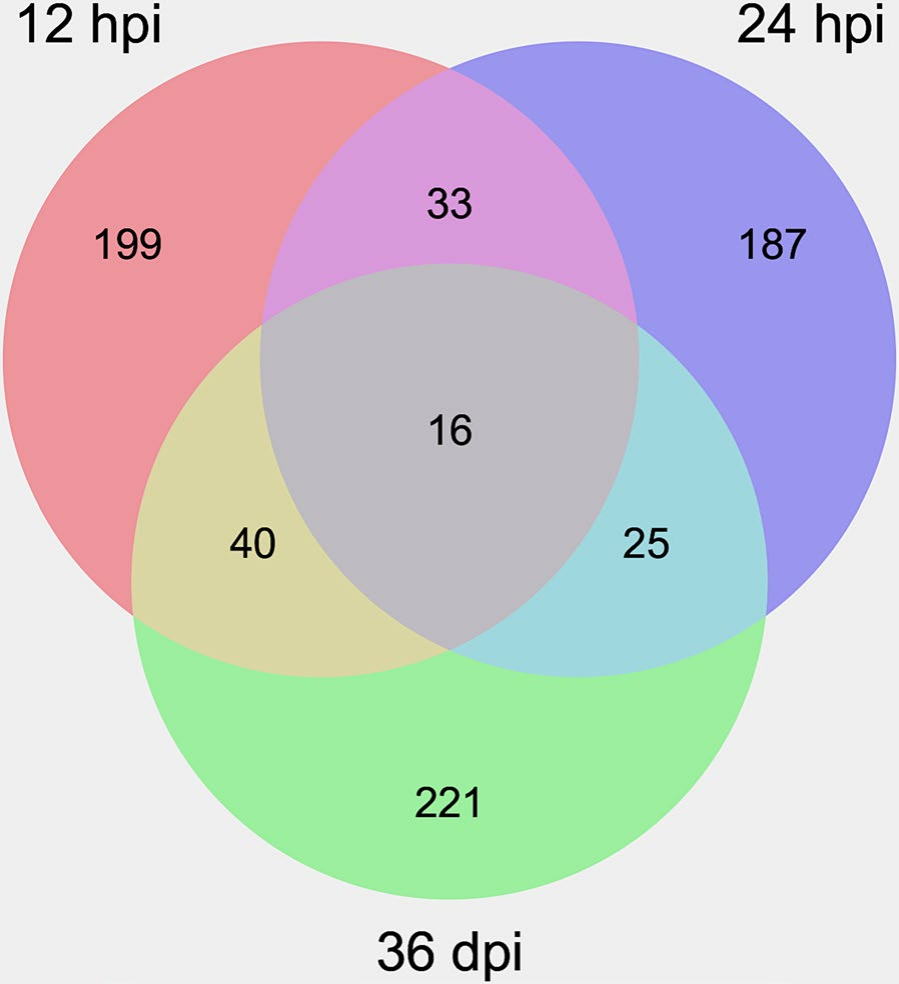
Venn diagram of the differentially expressed mRNAs at 12 hpi, 24 hpi and 36 dpi

Compared to the control groups, a total of 876 DElncRNAs and 288 DEmRNAs were identified at 12 hpi; 906 DElncRNAs and 261 DEmRNAs were identified at 24 hpi; 876 DElncRNAs and 302 DEmRNAs were identified at 36 dpi (Fig. 1 and Additional file 1: Table S1). Also, 16 DEmRNAs (e.g. *crp*, *dpp4* and *gnas*) were commonly detected at the three infection stages (Fig. 2 and Table 3), of which the expression level of C-reactive protein (*crp*) was significantly downregulated at 24 hpi and 36 dpi. In addition, the expression of dipeptidyl peptidase 4 (*dpp4*) was significantly upregulated at 12 hpi, reaching its maximum at 24 hpi, but was significantly downregulated at 36 dpi. The expression of G-protein alpha-subunit (*gnas*) was significantly increased at 24 hpi (Table 3). The expression profile of the DElncRNAs and DEmRNAs was confirmed by qRT-PCR (Additional file 2: Fig. S1).

**Table 3:** Common differentially expressed mRNAs in the dog’s liver at three stages post *Toxocara canis* infection

Transcript_id	Gene_name	Expression level		
		12 hpi	24 hpi	36 dpi
ENSCAFT00000022944	*map4k5*	Down	Down	Down
ENSCAFT00000018706	*crp*	Up	Down	Down
ENSCAFT00000029781	*slc25a14*	Down	Up	Down
ENSCAFT00000036718	*gnas*	Down	Up	Down
ENSCAFT00000011205	*ccdc92*	Down	Down	Up
ENSCAFT00000002620	*mtap*	Up	Down	Up
ENSCAFT00000046782	*fermt2*	Down	Down	Down
ENSCAFT00000024539	*kcnma1*	Up	Down	Up
ENSCAFT00000024868	*stx18*	Up	Down	Down
ENSCAFT00000044386	*ccdc158*	Up	Down	Up
ENSCAFT00000044024	*dpp4*	Up	Up	Down
ENSCAFT00000048979	*cxadr*	Up	Up	Down
ENSCAFT00000029120	*colec12*	Down	Down	Down
ENSCAFT00000043860	-	Down	Down	Down
ENSCAFT00000021794	*tet1*	Up	Up	Up
ENSCAFT00000007699	*gab2*	Down	Up	Up

### GO annotation and KEGG pathway analysis of the DEmRNAs

A total of 482, 744 and 610 GO terms were significantly enriched at 12 hpi, 24 hpi and 36 dpi, respectively (Additional file 3: Table S2). At 12 hpi, most of the DEmRNAs were mainly associated with cellular processes (GO:0000902, GO:0016043, GO:0071840 and GO:0032989); at 24 hpi, DEmRNAs were mainly related to lipoprotein component (GO:0034364, GO:0034358 and GO:0032994); at 36 dpi, DEmRNAs were mainly involved in the regulation of growth of symbiont involved in interaction with host (GO:0044130, GO:0044146, GO:0044126, GO:0044144, GO:0044110, GO:0044116 and GO:0044117) (Additional file 3: Table S2).

Some GO terms related to immune response or inflammation were observed at three stages of infection (Additional file 4: Table S3 and Additional file 5: Fig. S2). At 12 hpi, 47 DEmRNAs were significantly enriched in 44 immune- or inflammation-related GO terms, and the top 30 terms were mainly associated with immune system process (GO:0002376), negative regulation of immune system process (GO:0002683), Toll-like receptor 3 signaling pathway (GO:0034138) and cell adhesion-mediated by integrin (GO:0033627) (Additional file 5: Fig. S2a). At 24 hpi, 49 DEmRNAs were significantly enriched in 47 immunity- or inflammation-related GO terms, such as immune system process (GO:0002376), immune response (GO:0006955), immune system development (GO:0002520), cytokine production (GO:0001816) and T cell activation (GO:0042110) (Additional file 5: Fig. S2b).

**Fig. 3:**
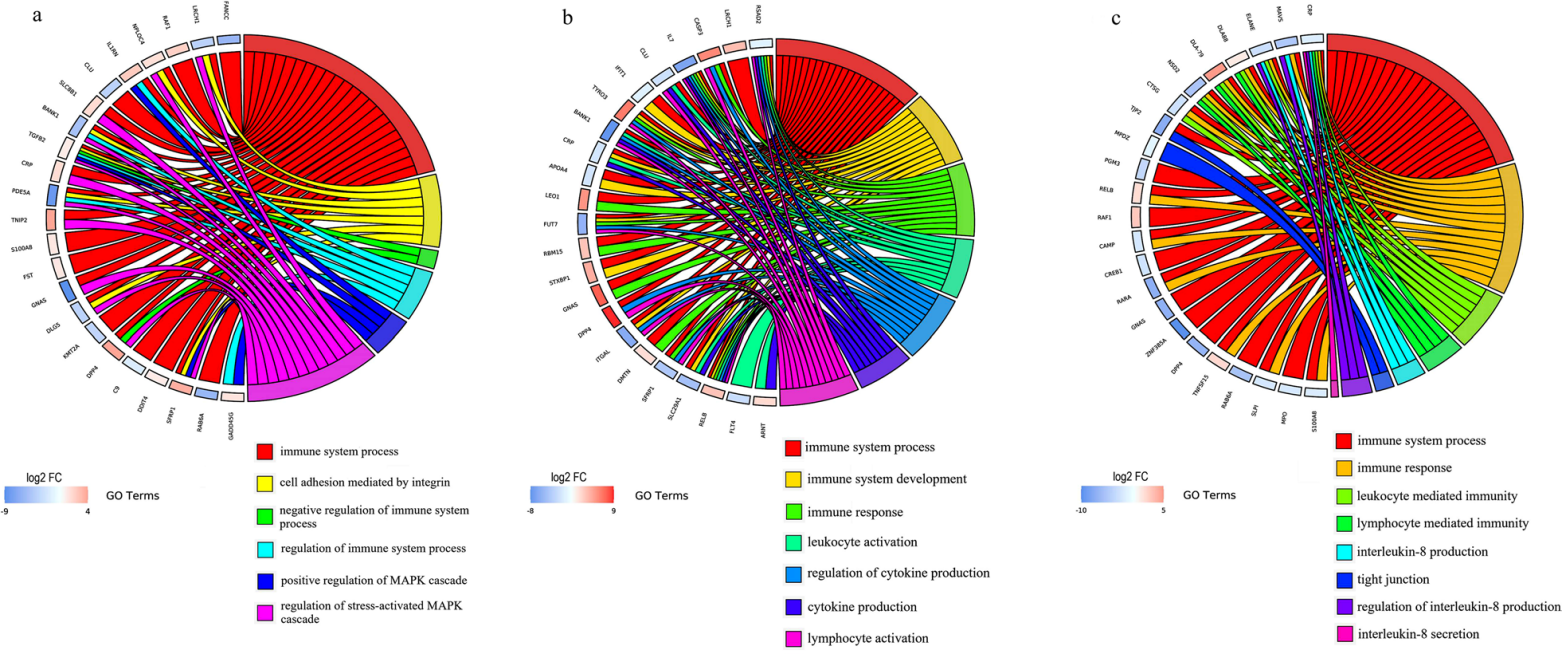
Chord diagrams showing the gene ontology (GO) of the differentially expressed genes in the liver at 12 hpi (**a**), 24 hpi (**b**) and 36 dpi (**c**), respectively. The diagrams show the clustered genes and their assigned immune- or inflammation-related GO terms, which are connected by ribbons. On the left side of the chord plot, blue to red represents the log2 fold change. On the right side of the chord plot, different colors ranging from red to pink represent different GO terms

At 36 dpi, 45 DEmRNAs were significantly enriched in 63 immune- or inflammation-related GO terms that were mainly associated with leukocyte-mediated cytotoxicity (GO:0001909), antigen processing and presentation (GO:0019882) and regulation of leukocyte-mediated cytotoxicity (GO:0001910), and interleukin-8 production (GO:0032637) (Additional file 5: Fig. S2c). In addition, DEmRNA *crp* was significantly enriched in immune system process (GO:0002376), regulation of stress-activated MAPK cascade (GO:0032872), immune system development (GO:0002520), leukocyte mediated immunity (GO:0002443), cytokine production (GO:0001816), immune response (GO:0006955), leukocyte-mediated immunity (GO:0002443), lymphocyte-mediated immunity (GO:0002449), interleukin-8 production (GO:0032637), regulation of interleukin-8 production (GO:0032677) and interleukin-8 secretion (GO:0072606). The *dpp4* was involved in the immune system process (GO:0002376), regulation of cytokine production (GO:0001817) and lymphocyte activation (GO:0046649) at three stages of infection (Fig. 3a–c).

**Fig. 4:**
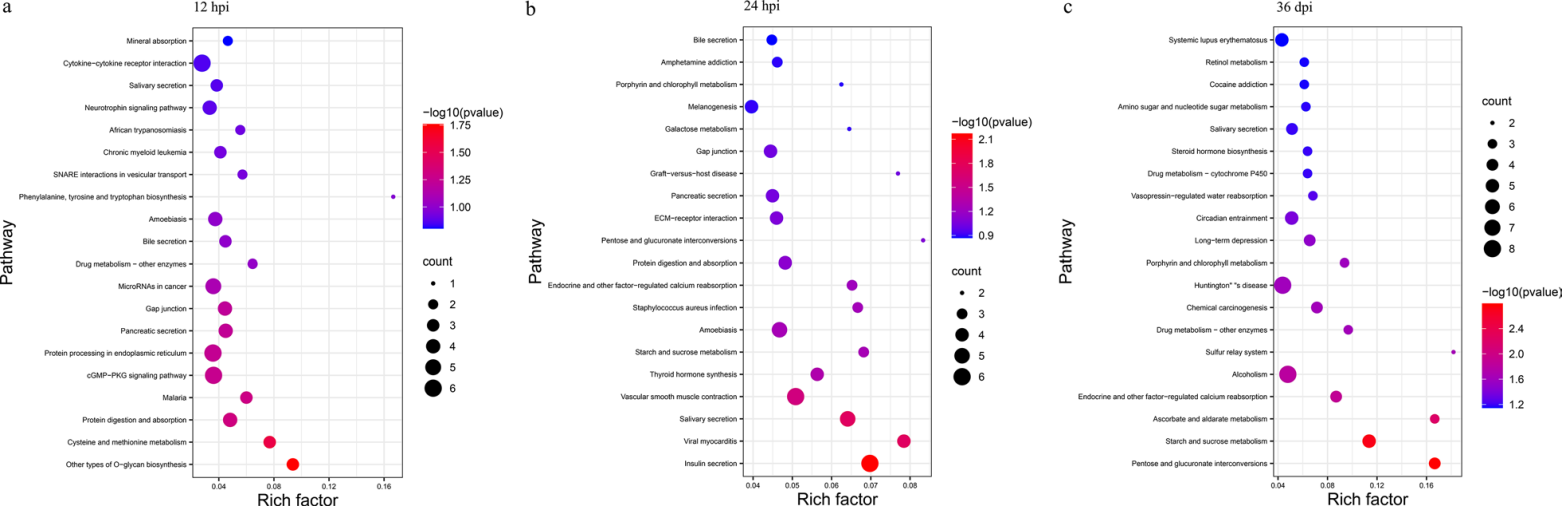
Scatter plots showing KEGG pathway enrichment results of the differentially expressed (DE) mRNAs. The top 20 enriched pathways at 12 hpi (**a**), 24 hpi (**b**) and 36 dpi (**c**), respectively. The X-axis labels denote an enrichment score (-log_10_ *P*-value), and the Y-axis labels represent the names of KEGG pathways. The rich factor represents the number of DEmRNAs enriched in a pathway to the total number of annotated genes in that pathway. The greater the value of the rich factor is, the greater the degree of KEGG pathway enrichment

The KEGG enrichment analysis at 12 hpi revealed that 10 DEmRNAs (e.g. *tat*) were significantly enriched in three pathways, including other types of O-glycan biosynthesis, cysteine and methionine metabolism, and protein digestion and absorption (Fig. 4a). At 24 hpi, 13 DEmRNAs (e.g. *gnas*, *itgal*) were significantly enriched in five pathways, including insulin secretion, viral myocarditis, salivary secretion, vascular smooth muscle contraction and thyroid hormone synthesis (Fig. 4b). At 36 dpi, 27 DEmRNAs (e.g. *hap1*, *clta* and *dctn4*) were significantly enriched in 12 pathways, including pentose and glucuronate interconversions, starch and sucrose metabolism, ascorbate and aldarate metabolism, endocrine and other factor-regulated calcium reabsorption, alcoholism, sulfur relay system, drug metabolism-other enzymes, chemical carcinogenesis, Huntington's disease, porphyrin and chlorophyll metabolism, long-term depression and circadian entrainment (Fig. 4c and Additional file 6: Table S4).

### Co-localization of DElncRNAs

At 12 hpi, 63 DElncRNAs were located in the vicinity of 47 DEmRNAs. Among these DElncRNAs, LNC_015175, LNC_015166, LNC_015177, LNC_015172, LNC_015165 and LNC_015176 were located in the vicinity of the *c9* gene. The LNC_008755 and LNC_010262 were located in the vicinity of *fbxl2* and *atg101* genes, respectively. The transcriptional level of LNC_001408 located in the vicinity of the *il1r1* and *il1rl1* gene was upregulated. The LNC_001412 was located in the vicinity of the *il1r2* and was downregulated (Additional file 7: Table S5 and Additional file 8: Fig. S3a).

At 24 hpi, 73 DElncRNAs were found in the adjacent area of 55 DEmRNAs. The LNC_015756 was located in the adjacent area of the apolipoprotein A4 (*apoa4*) and *il1a* genes and significantly downregulated (Additional file 8: Fig. S3b). Also, the LNC_008220, LNC_008226, LNC_008050 and LNC_008052, which were located in the vicinity of the *saa1* gene, were significantly decreased at 24 hpi and 36 dpi. The expression of LNC_006670, located in the vicinity of *nod2* gene and *cyld* gene, was significantly upregulated. The LNC_002735 is located with the adjacency of the *btnl2* gene (Additional file 8: Fig. S3b, c).

At 36 dpi, 58 DElncRNAs were found within the up- or downstream of 52 DEmRNAs (Additional file 7: Table S5 and Additional file 8: Fig. S3), including the LNC_013524 that was located in the vicinity of *il1rap* gene, and were significantly downregulated. The expression levels of LNC_005671 and LNC_011661, which were located with in the vicinity of *trim44* and *otop1*, respectively, were significantly upregulated. The LNC_007354 was located in the vicinity of the *tlr9* gene and was significantly upregulated. The transcription levels of LNC_011050 and LNC_011052, which were located in the vicinity of the *pdcd4* gene, were significantly increased. The LNC_005105 and LNC_005401 were located in the vicinity of the *il1rn* gene and were significantly up- and downregulated at 36 dpi, respectively (Additional file 8: Fig. S3c).

### GO annotation analysis of DElncRNAs

**Fig. 5:**
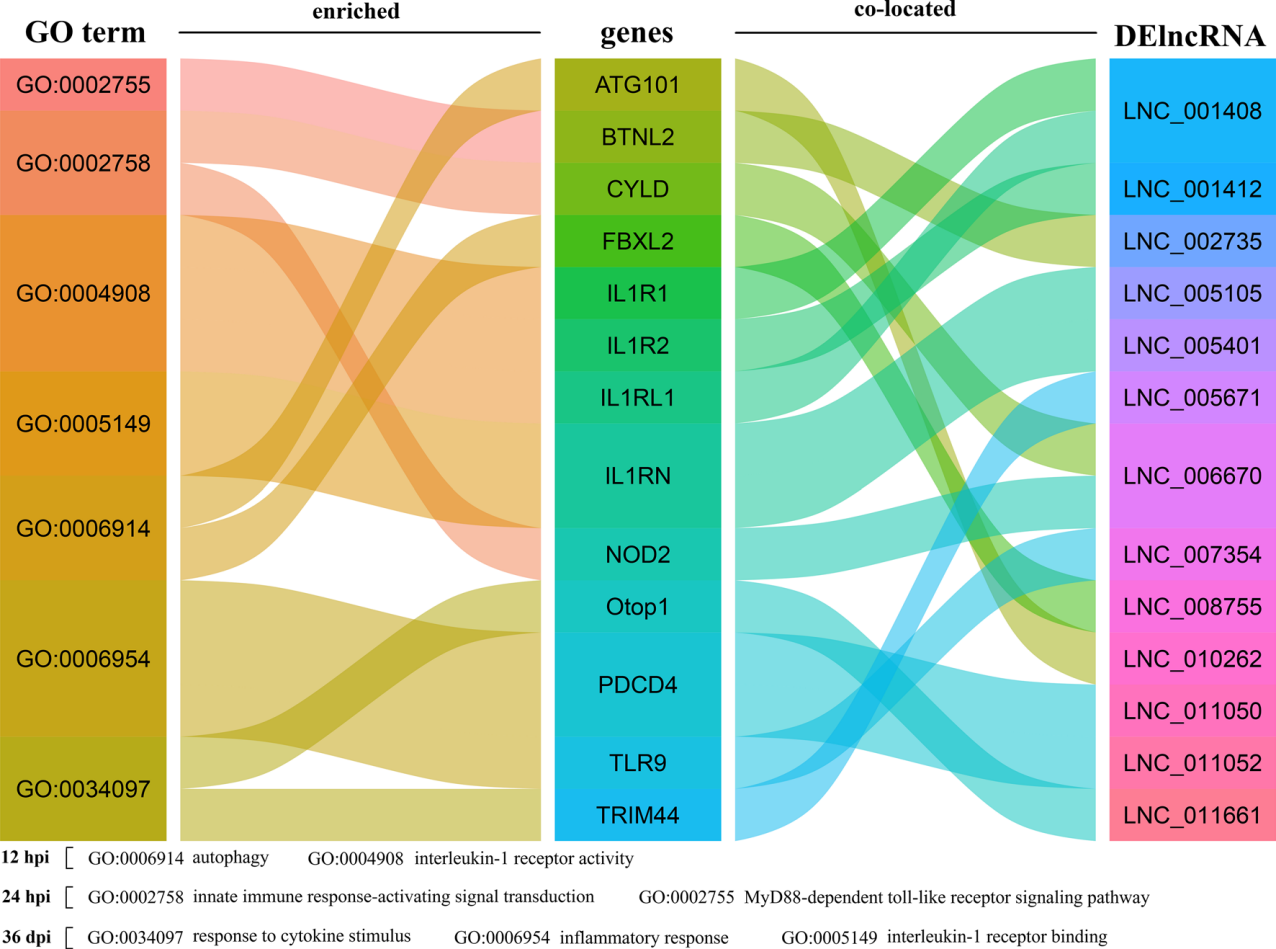
Alluvial plots showing partial immune- or inflammation-related GO terms of target genes of the differentially expressed (DE) lncRNAs at three stages of infection. The GO terms, target genes and DElncRNAs are shown on the left, middle and right side of the plot, respectively

GO enrichment analyses of the potential target genes (co-localization) revealed that 482, 744 and 610 GO terms were significantly enriched at 12 hpi, 24 hpi and 36 dpi, respectively (Additional file 9: Table S6). In addition, some immune- or inflammation-related terms of DElncRNAs were observed at three infection stages, and the top 30 GO terms are shown in Additional file 10: Fig. S4. Among those GO terms, at 12 hpi, 156 target mRNAs (e.g. *fbxl2* and *atg101*) of DElncRNAs (e.g. 181 DElncRNAs) were significantly enriched in 24 immune- or inflammation-related GO terms (e.g. autophagy) (Fig. 5). At 24 hpi, 178 target mRNAs (e.g. *nod2* and *cyld*) of DElncRNAs (including 188 DElncRNAs) were significantly enriched in 68 immune- or inflammation-related GO terms (e.g. innate immune response process). At 36 dpi, 428 target mRNAs (e.g. trim44, *otop1*, *tlr9* and *pdcd4*) of DElncRNAs (e.g. 367 DElncRNAs) were significantly enriched in 42 immune- or inflammation-related GO terms (e.g. response to cytokine stimulus and inflammatory response) (Additional file 11: Table S7).

## Discussion

Genomics, transcriptomics and proteomics approaches have been previously used to obtain insight into the pathogenesis of *T. canis* [9]. In the present study, we profiled the changes in the expression of mRNAs and lncRNAs in the liver of Beagle dogs during *T. canis* infection.

### Function of the DEmRNAs at three infection stages

GO enrichment analysis showed that many DEmRNAs were related to immune processes at the early stages of infection (12 and 24 hpi). However, at the late stage of infection (36 dpi), many DEmRNAs were related to leukocyte-mediated processes. This difference may be related to the migration of the larvae in Beagle dogs during *T. canis* infection. The *crp* is a marker of inflammation and tissue damage and is involved in the immune process and regulation of interleukin-8 (*il-8*) [32]. The downregulation of *crp* at 36 dpi suggests that *T. canis* reduces inflammation in the dog’s liver at the late stage of infection. The *crp* has been used to monitor the development of heartworm disease in dogs [33], suggesting that the abnormal expression of *crp* may be used as a potential biomarker for the early detection of toxocariasis. In the present study, *dpp4* was significantly upregulated at the early stages of infection. The *dpp4* is ubiquitously expressed in the liver and other tissues and is considered a co-stimulatory molecule to control the differentiation and immune responses of T cells [34]. These findings suggest that *T. canis* could induce the immune responses of the dog liver at the early infection stage, and *dpp4* may be associated with immune response in the liver.

KEGG analysis showed that *tat* was significantly increased at 12 hpi and was involved in cysteine and methionine metabolism pathways. Methionine is an essential amino acid, which regulates the metabolic processes, innate immune system and digestive function in mammals [35]. This suggests that *tat* may modulate the innate immune response of livers in dogs infected by *T. canis*. However, the exact role of *tat* in the hepatic innate immune process during *T. canis* infection remains to be investigated. *gnas* and *itgal* were enriched in the insulin secretion pathway and viral myocarditis pathway, respectively, at 24 hpi. Increasing evidence suggests that the immune system can play a crucial role in tuning metabolic homeostasis. For example, the immune system regulates the endocrine function of islets of Langerhans cells [36] and can stimulate the production of beta-cell insulin under steady state [36]. The *gnas*-activating mutations can form a rare subgroup of inflammatory liver tumors [37]. Among the gastric foveolar metaplasia lesions, *gnas* mutations are more common in lesions without active inflammation [38]. The *gnas* was significantly increased at 24 hpi, suggesting that *gnas* is likely involved in the liver inflammatory and immune response induced by *T. canis* infection. Myocarditis is caused by viral infections and/or post-viral immune-mediated responses [39]. The *itgal* is related to inflammation and apoptosis [40] and was significantly downregulated at 24 hpi. These findings indicate that *itgal* plays a role in the inflammatory reactions in the liver during *T. canis* infection. In this study, *hap1*, *clta* and *dctn4* genes were significantly upregulated and related to Huntington's disease pathway at 36 dpi. Huntington's disease is an autosomal-dominant neurodegenerative disease caused by the expansion of CAG repeats in the *Huntingtin* (*htt*) gene [41]. Mutant huntingtin protein (mHTT) can promote cell-autonomous immune activation when it is highly expressed in immune cells [42]. Inflammation has been suggested as an important feature of Huntington’s disease [43]. Hence, we speculate that these genes in the puppies’ livers may promote immune and inflammatory responses of puppies to *T. canis* infection.

### Co-localization analysis of DElncRNAs and DEmRNAs

We detected multiple mRNAs whose regulation was mediated by several lncRNAs at all three infection stages, suggesting a complex regulatory relationship between DElncRNAs and DEmRNAs (Additional file 7: Table S5). The complement *c9* plays a crucial role in the innate immune response to pathogens [44]. The *c9* is the major component to form a pore-like structure, which is characteristic of the fully formed membrane attack complex [45]. The membrane attack complex forms on the cell membrane and plays an important role in innate immune response [46]. From the DElncRNA-mRNA co-located network, we found the target gene *c9* was regulated by LNC_015175, LNC_015166 and LNC_015177. The target gene *c9* was significantly decreased at 12 hpi, as well as the transcriptional level of LNC_015175, LNC_015166 and LNC_015177. These results reveal that these DElncRNAs might be associated with the immune response of the liver to *T. canis* infection at an early stage.

In this study, LNC_015756 was located in the vicinity of *apoa4* gene and was significantly downregulated at 24 hpi. *apoa4* is a member of the apolipoprotein family of lipid-binding proteins, which is involved in the regulation of the immune responses [47–49]. *apoa4* reduces the secretion of pro-inflammatory cytokines in human peripheral blood mononuclear cells [50], suggesting that *apoa4* may be involved in an anti-inflammatory feedback loop. It was also considered an anti-inflammatory agent in the immune system. Another gene, *il-1α* (*IL1A*), migrates from the nucleus to the cytosol; when the cell is exposed to a necrotic signal, the lysates of these cells are highly inflammatory [51]. *il-1α* also evokes significant inflammatory reactions via the IL-1R1 from necrotic cells [52]. The transcriptional level of *il1a* was significantly increased at 24 hpi and regulated by LNC_015756. These findings suggest that LNC_015756 may play a pro-inflammatory role in the liver of puppies to counter *T. canis* infection. Serum amyloid A (*saa*) includes *saa1*, *saa2*, *saa3* and *saa4*, which can be used as a diagnostic or prognostic marker for many diseases [53, 54]. Each of the *saa* genes encodes a different protein such as *saa1*-*4*, of which *saa1* and *saa2* are induced by inflammatory cytokines. When an infectious or inflammatory stimulus persists, the liver produces more *saa* to counter infection [53]. The *saa1* was significantly downregulated and regulated by LNC_008220, LNC_008226, LNC_008050 and LNC_008052 at 24 hpi and 36 dpi. We speculate that *T. canis* larvae reduce inflammatory cytokines at 24 hpi and 36 dpi to facilitate its migration in the liver by downregulating these LncRNAs and *saa1* gene. We also found that LNC_013524 regulates target gene *il1rap* at 36 dpi. *Il-1racp* (*IL1RAP*) and *il-33* play important roles in the innate immune response [55]. When *il-33* binds to ST2 and *il-1racp*, the *il-33* receptor complex is activated and recruits the adaptor molecule myeloid differentiation factor 88 (*MyD88*) [56]. *Myd88* plays an important role in early innate immune responses during coronavirus-induced encephalomyelitis [57]. *Il1rap* and the LNC_013524 were significantly downregulated at 36 dpi. LNC_013524 may participate in the innate immunity of liver during *T. canis* infection, and the downregulation of LNC_013524 may compromise the innate immune response, therefore increasing *T. canis* survival at a late stage of infection.

### Functions of DElncRNAs at three stages of infection

Autophagy is involved in multiple processes of innate and adaptive immune responses in mammalian cells, and with the help of autophagy adaptor proteins, it can trigger a series of immune responses through pattern recognition receptors (PRRs) [58]. These processes play an essential role in eliminating intracellular microorganisms [58]. Moreover, autophagy influences the adaptive immune response by regulating homeostasis of antigen-presenting cells [59]. In this study, the LNC_008755 and LNC_010262 were significantly downregulated and regulated *fbxl2* and *atg101* genes that were enriched in autophagy at 12 hpi. These findings suggest that LNC_008755 and LNC_010262 may be related to the hepatic immune responses to *T. canis* infection. The *il-1* family of ligands and receptors is related to acute and chronic inflammation. Anti-inflammatory members of the *il-1* family can affect disease severity [60]. The *il1r1*, *il1rl1* and *il1r2* were regulated by LNC_001408 and LNC_001412, which participate in interleukin-1 receptor activity term at 12 hpi. LNC_001408 was significantly increased and LNC_001412 significantly decreased at 12 hpi. These findings indicate that expression of LNC_001408 and LNC_001412 may be related to *T. canis* infection-triggered inflammation in puppy liver at an early stage of *T. canis* infection.

Notably, both *nod2* and *cyld* were enriched in the immune response process and could be regulated by LNC_006670. *nod1* and *nod2* play fundamental and pleiotropic roles in host defense against infection [61]. CYLD lysine 63 deubiquitinase (*cyld*) plays a crucial role in regulating IFN receptor signaling [62]. LNC_006670 was significantly increased at 24 hpi. Hence, we speculate that upregulation of LNC_006670 in the liver may promote the immune response of puppies against *T. canis* infection via regulating the expression of *nod2* and *cyld*. Also, *btnl2* was regulated by LNC_002735 and was enriched in the *MyD88*-dependent Toll-like receptor signaling pathway at 24 hpi. During *Trypanosoma cruzi* infection, *MyD88* plays an important role in the development of a robust Th1 response [63]. *MyD88* is an elementary adaptor molecule for most Toll-like receptors (TLRs), which can mediate the induction of inflammatory cytokines via nuclear factor κB (NF-κB) [64]. TLRs can also mediate infection-induced inflammation and sterile inflammation via endogenous molecules [65]. *Btnl2*, as a negative co-stimulatory molecule, has an important role in inflammatory disease [66]. LNC_006670 was significantly downregulated at 24 hpi, indicating that downregulation of LNC_002735 may regulate *btnl2* to mediate the inflammatory response of the puppies’ liver.

At 36 dpi, the target genes *trim44* and *otop1* were regulated by LNC_005671 and LNC_011661, respectively, and were enriched in the response to cytokine stimulus. Cytokines mediate and control the inflammatory and immune responses [67]. *Trim44* regulates the virus-triggered immune response via enhancing the stability of VISA [68]. *Otop1* (Otopetrin 1) as a unique target of cytokine signaling can reduce the adipose tissue inflammation caused by obesity [69]. LNC_005671 and LNC_011661 were significantly upregulated at 36 dpi, suggesting that upregulation of these lncRNAs could enhance immune response and weaken tissue inflammation against *T. canis* infection in the puppies’ liver at late stages of infection. *Tlr9* and *pdcd4* were enriched in the inflammatory response and were regulated by LNC_007354, LNC_011050 and LNC_011052, respectively. *Tlr9* activates inflammatory factors such as *il-1* and *il-8*, increasing immune inhibitory factor *il-10* secretion and MMP-2 expression to enhance the invasion and metastasis of lung cancer cells [70, 71]. The LNC_007354 was significantly upregulated at 36 dpi. LNC_007354 regulates *tlr9* to activate pro-inflammatory factors and increases immune inhibitory factors to promote the invasion and migration of *T. canis* larvae. *Pdcd4* (programmed cell death 4) may suppress the activation of inflammatory macrophages via NF-kB and mitogen-activated protein kinase (MAPK) signaling in atherosclerosis [72]. The deficiency of *pdcd4* significantly increases the level of pro-inflammatory cytokines, such as *il-6* and lipopolysaccharide/D-galactosamine, leading to acute liver injury, colitis and colorectal cancer [73, 74]. LNC_011050 and LNC_011052 were significantly upregulated at 36 dpi, suggesting that they may play roles in suppressing pro-inflammatory cytokines and liver injury repair post *T. canis* infection.

The *il1rn* was enriched in the interleukin-1 receptor binding and was regulated by LNC_005105 and LNC_005401, which were significantly up- and downregulated at 36 dpi, respectively. The mutation of *il1rn* (interleukin 1 receptor antagonist) can lead to a deficiency of the *il-1*-receptor antagonist causing an autosomal recessive autoinflammatory disease [75]. The *il1rn* gene encodes interleukin-1 receptor antagonist (*il-1ra*), which is a potent anti-inflammatory cytokine that competitively inhibits stimulation by inflammatory mediators via binding to *il-1r1* and preventing the recruitment of *il-1racp* [76]. The altered expression of LNC_005105 and LNC_005401 may be associated with the release of anti-inflammatory cytokines that mediate the healing of the liver pathology at the late stage of infection.

## Conclusions

We performed a comprehensive analysis of lncRNA and mRNA expression patterns in the liver of Beagle dogs infected by *T. canis* at three stages after infection. The functional enrichment analysis showed that many DEmRNAs are associated with the immune responses of the puppies’ liver at an early stage of infection. Co-localization analysis suggested that some DElncRNAs might play an anti-inflammatory role in in the liver during *T. canis* infection. These findings provided new insight into the transcriptomic basis of the interaction between *T. canis* and the definitive canine host. Future studies should focus on investigation of the exact roles of the identified DElncRNAs and miRNAs in the innate/adaptive immune response of dogs to *T. canis* infection.

## Supplementary Information

**Additional file 1: Table S1.** The differentially expressed mRNAs and lncRNAs in the liver of puppies at three stages post *Toxocara canis* infection.

**Additional file 2: Figure S1.** The qRT-PCR confirmation of the differentially expressed lncRNAs and mRNAs identified by RNA-Seq. The red and gray columns show the fold change values obtained by RNA sequencing and qRT-PCR, respectively. Y-axis shows the relative change of lncRNA and mRNA levels expressed as fold increase compared with the control *L13A*. The X-axis shows the name of the 10 RNAs used in the analysis.

**Additional file 3: Table S2.** GO terms of the differentially expressed mRNAs in the liver of puppies at three stages post *Toxocara canis* infection.

**Additional file 4: Table S3.** Immune- or inflammation-related GO terms of the differentially expressed mRNAs at three stages post *Toxocara canis* infection.

**Additional file 5: Figure S2.** The top 30 immune- or inflammation-related GO terms of the differentially expressed (DE) mRNAs. The significantly enriched GO terms in biological process (red column), cellular component (blue column) and molecular function (green column) at (**a**) 12 hpi, 24 hpi (**b**) and 36 dpi (**c**). The left Y-axis shows the number of DEmRNAs. The X-axis shows the name of the GO terms.

**Additional file 6: Table S4.** KEGG pathways of the differentially expressed mRNAs at three stages post *Toxocara canis* infection.

**Additional file 7: Table S5.** The co-localization of the mRNAs of the differentially expressed lncRNAs at three stages post *Toxocara canis* infection.

**Additional file 8: Figure S3.** The co-localization networks of DElncRNAs and DEmRNAs at (**a**) 12 hpi, (**b**) 24 hpi and (**c**) 36 dpi, respectively. The diamonds represent the DElncRNAs and circles represent the DEmRNAs. The up- and down-regulated RNAs are indicated by red and blue colors, respectively.

**Additional file 9: Table S6.** GO terms of the differentially expressed lncRNAs in the liver of puppies at three stages of infection.

**Additional file 10: Figure S4.** The top 30 immune- or inflammation-related GO terms of the differentially expressed (DE) lncRNAs. The significantly enriched GO terms in biological process (red column), cellular component (blue column) and molecular function (green column) terms at 12 hpi (**a**), 24 hpi (**b**) and 36 dpi (**c**). The left Y-axis shows the number of DElncRNAs. The X-axis shows the name of the GO terms.

**Additional file 11: Table S7.** Immune- or inflammation-related GO terms of the differentially expressed lncRNAs in the liver of puppies at three stages post *Toxocara canis* infection.

## Data Availability

The datasets supporting the findings of this article are included within the article and its additional files. The RNA-Seq raw data are available in the NCBI SRA repository under accession number PRJNA649207.

## Abbreviations

*T. **canis*: *Toxocara canis*
lncRNAs: Long non-coding RNAs
DElncRNAs: Differentially expressed lncRNAs
DEmRNAs: Differentially expressed mRNAs
hpi: Hours post-infection
dpi: Days post-infection
*dpp4*: Dipeptidyl peptidase-4
*crp*: C-reactive protein
*gnas*: G-protein alpha-subunit
RIN: RNA integrity number
bp: Base pair
FPKMs: Fragments per kilobase of exon model per million fragments
CNCI: Coding-non-coding-index
CPC: Coding potential calculator
GO: Gene ontology
BP: Biological process
CC: Cellular component
MF: Molecular function
KEGG: Kyoto Encyclopedia of Genes and Genomes
qRT-PCR: Quantitative real-time PCR
ceRNA: Competing endogenous RNA
MAPK: Mitogen-activated protein kinase
*itgal*: Integrin subunit alpha L
*tat*: Tyrosine aminotransferase
*hap1*: Huntingtin associated protein 1
*clta*: Clathrin light chain A
*dctn4*: Dynactin subunit 4
*fbxl2*: F-Box and leucine-rich repeat protein 2
*atg101*: Autophagy related 101
*il1r1*: Interleukin 1 receptor type 1
*il1rl1*: Interleukin 1 receptor like 1
*apoa4*: Apolipoprotein A4
*il1a*: Interleukin 1 alpha
*nod2*: Nucleotide-binding oligomerization domain containing 2
*cyld*: CYLD lysine 63 deubiquitinase
*btnl2*: Butyrophilin-like 2
*il1rap*: Interleukin 1 receptor accessory protein
*trim44*: Tripartite motif containing 44
*otop1*: Otopetrin 1
*pdcd4*: Programmed cell death 4
*il1rn*: Interleukin 1 receptor antagonist
il-8: Interleukin-8
*htt*: *Huntingtin*
mHTT: Mutant huntingtin protein
*c9*: Complement C9
PBMCs: Peripheral blood mononuclear cells
*saa*: Serum amyloid A
*Myd88*: Myeloid differentiation factor 88
PRRs: Pattern recognition receptors
TLRs: Toll-like receptors
NF-κB: Nuclear factor κB

## References

[1] Wolfe A, Wright IP (2003). Human toxocariasis and direct contact with dogs. Vet Rec.

[2] Schnieder T, Laabs EM, Welz C (2011). Larval development of *Toxocara canis* in dogs. Vet Parasitol.

[3] Ma G, Holland CV, Wang T, Hofmann A, Fan CK, Maizels RM (2018). Human toxocariasis. Lancet Infect Dis.

[4] Zheng WB, Zou Y, Liu GH, Zhu XQ (2020). Epidemiology of *Toxocara spp* in dogs and cats in mainland China, 2000–2019. Adv Parasitol..

[5] Buijs J, Egbers MW, Lokhorst WH, Savelkoul HF, Nijkamp FP (1995). *Toxocara*-induced eosinophilic inflammation airway function and effect of anti-IL-5. Am J Respir Crit Care Med.

[6] Kayes SG (1986). Nonspecific allergic granulomatosis in the lungs of mice infected with large but not small inocula of the canine ascarid, *Toxocara canis*. Clin Immunol Immunopathol.

[7] Pinelli E, Withagen C, Fonville M, Verlaan A, Dormans J, van Loveren H (2005). Persistent airway hyper-responsiveness and inflammation in *Toxocara canis*-infected BALB/c mice. Clin Exp Allergy.

[8] Zou Y, Zheng WB, He JJ, Elsheikha HM, Zhu XQ, Lu YX (2020). *Toxocara canis* differentially affects hepatic microRNA expression in beagle dogs at different stages of infection. Front Vet Sci.

[9] Zheng WB, Zou Y, Zhu XQ, Liu GH (2020). *Toxocara* “omics” and the promises it holds for medicine and veterinary medicine. Adv Parasitol.

[10] Xu Y, Zheng WB, Li HY, Cai L, Zou Y, Xie SC (2022). RNA sequencing reveals dynamic expression of spleen lncRNAs and mRNAs in Beagle dogs infected by *Toxocara canis*. Parasit Vectors.

[11] Zheng WB, Zou Y, He JJ, Elsheikha HM, Liu GH, Hu MH (2021). Global profiling of lncRNAs-miRNAs-mRNAs reveals differential expression of coding genes and non-coding RNAs in the lung of beagle dogs at different stages of *Toxocara canis* infection. Int J Parasitol.

[12] Rinn JL, Chang HY (2012). Genome regulation by long noncoding RNAs. Annu Rev Biochem.

[13] Guil S, Esteller M (2012). Cis-acting noncoding RNAs: friends and foes. Nat Struct Mol Biol.

[14] Fatica A, Bozzoni I (2014). Long non-coding RNAs: new players in cell differentiation and development. Nat Rev Genet.

[15] Zhou R, Feng Y, Chen XM (2014). Non-coding RNAs in epithelial immunity to *Cryptosporidium* infection. Parasitology.

[16] Bayer-Santos E, Marini MM, da Silveira JF (2017). Non-coding RNAs in host-pathogen interactions: subversion of mammalian cell functions by protozoan parasites. Front Microbiol.

[17] Ren GJ, Fan XC, Liu TL, Wang SS, Zhao GH (2018). Genome-wide analysis of differentially expressed profiles of mRNAs, lncRNAs, and circRNAs during *Cryptosporidium baileyi* infection. BMC Genomics.

[18] Zheng WB, Zou Y, Elsheikha HM, Liu GH, Hu MH, Wang SL (2019). Serum metabolomic alterations in beagle dogs experimentally infected with *Toxocara canis*. Parasit Vectors.

[19] Song F, Wang L, Zhu W, Dong Z (2019). Long noncoding RNA and mRNA expression profiles following igf3 knockdown in common carp. Cyprinus carpio Sci Data.

[20] Zhou J, Xiong Q, Chen H, Yang C, Fan Y (2017). Identification of the spinal expression profile of non-coding RNAs involved in neuropathic pain following spared nerve injury by sequence analysis. Front Mol Neurosci.

[21] Langmead B, Salzberg SL (2012). Fast gapped-read alignment with Bowtie 2. Nat Methods.

[22] Pertea M, Kim D, Pertea GM, Leek JT, Salzberg SL (2016). Transcript-level expression analysis of RNA-seq experiments with HISAT. StringTie and Ballgown Nat Protoc.

[23] Trapnell C, Williams BA, Pertea G, Mortazavi A, Kwan G, van Baren MJ (2010). Transcript assembly and quantification by RNA-Seq reveals unannotated transcripts and isoform switching during cell differentiation. Nat Biotechnol.

[24] Sun L, Luo H, Bu D, Zhao G, Yu K, Zhang C (2013). Utilizing sequence intrinsic composition to classify protein-coding and long non-coding transcripts. Nucleic Acids Res.

[25] Kong L, Zhang Y, Ye ZQ, Liu XQ, Zhao SQ, Wei L (2007). CPC: assess the protein-coding potential of transcripts using sequence features and support vector machine. Nucleic Acids Res.

[26] Bateman A, Birney E, Cerruti L, Durbin R, Etwiller L, Eddy SR (2002). The Pfam protein families database. Nucleic Acids Res.

[27] Love MI, Huber W, Anders S (2014). Moderated estimation of fold change and dispersion for RNA-seq data with DESeq2. Genome Biol.

[28] Young MD, Wakefield MJ, Smyth GK, Oshlack A (2010). Gene ontology analysis for RNA-seq: accounting for selection bias. Genome Biol.

[29] Wu J, Mao X, Cai T, Luo J, Wei L (2006). KOBAS server: a web-based platform for automated annotation and pathway identification. Nucleic Acids Res.

[30] Lotia S, Montojo J, Dong Y, Bader GD, Pico AR (2013). Cytoscape app store. Bioinformatics.

[31] Livak KJ, Schmittgen TD (2001). Analysis of relative gene expression data using real-time quantitative PCR and the 2(-Delta Delta C(T)) Method. Methods.

[32] Kim JH (2020). Interleukin-8 in the tumor immune niche: lessons from comparative oncology. Adv Exp Med Biol.

[33] Carretón E, Morchón R, Montoya-Alonso JA (2017). Cardiopulmonary and inflammatory biomarkers in heartworm disease. Parasit Vectors.

[34] Klemann C, Wagner L, Stephan M, von Hörsten S (2016). Cut to the chase: a review of CD26/dipeptidyl peptidase-4's (DPP4) entanglement in the immune system. Clin Exp Immunol.

[35] Martínez Y, Li X, Liu G, Bin P, Yan W, Más D (2017). The role of methionine on metabolism, oxidative stress, and diseases. Amino Acids.

[36] Dalmas E (2019). Innate immune priming of insulin secretion. Curr Opin Immunol.

[37] Ding H, Zhang X, Su Y, Jia C, Dai C (2020). GNAS promotes inflammation-related hepatocellular carcinoma progression by promoting STAT3 activation. Cell Mol Biol Lett.

[38] Matsubara A, Ogawa R, Suzuki H, Oda I, Taniguchi H, Kanai Y (2015). Activating GNAS and KRAS mutations in gastric foveolar metaplasia, gastric heterotopia, and adenocarcinoma of the duodenum. Br J Cancer.

[39] Kindermann I, Barth C, Mahfoud F, Ukena C, Lenski M, Yilmaz A (2012). Update on myocarditis. J Am Coll Cardiol.

[40] Fraser L, Brym P, Pareek CS, Mogielnicka-Brzozowska M, Paukszto Ł, Jastrzębski JP (2020). Transcriptome analysis of boar spermatozoa with different freezability using RNA-Seq. Theriogenology.

[41] Siew JJ, Chen HM, Chen HY, Chen HL, Chen CM, Soong BW (2019). Galectin-3 is required for the microglia-mediated brain inflammation in a model of huntington’s disease. Nat Commun.

[42] Weiss A, Träger U, Wild EJ, Grueninger S, Farmer R, Landles C (2012). Mutant huntingtin fragmentation in immune cells tracks huntington’s disease progression. J Clin Invest.

[43] Colpo GD, Stimming EF, Rocha NP, Teixeira AL (2017). Promises and pitfalls of immune-based strategies for huntington’s disease. Neural Regen Res.

[44] Zheng H, Ji W, Zhang GR, Zhang XT, Shi ZC, Wei KJ (2016). Molecular characterization and expression analyses of the complement component C8α, C8β, and C9 genes in yellow catfish (Pelteobagrus fulvidraco) after the aeromonas hydrophila challenge. Int J Mol Sci.

[45] DiScipio RG, Berlin C (1999). The architectural transition of human complement component C9 to poly(C9). Mol Immunol.

[46] Dunkelberger JR, Song WC (2010). Complement and its role in innate and adaptive immune responses. Cell Res.

[47] Hegele RA (2009). Plasma lipoproteins: genetic influences and clinical implications. Nat Rev Genet.

[48] Chuang K, Elford EL, Tseng J, Leung B, Harris HW (2010). An expanding role for apolipoprotein E in sepsis and inflammation. Am J Surg.

[49] Zhang HL, Wu J, Zhu J (2010). The immune-modulatory role of apolipoprotein E with emphasis on multiple sclerosis and experimental autoimmune encephalomyelitis. Clin Dev Immunol.

[50] Recalde D, Ostos MA, Badell E, Garcia-Otin AL, Pidoux J, Castro G (2004). Human apolipoprotein A-IV reduces secretion of proinflammatory cytokines and atherosclerotic effects of a chronic infection mimicked by lipopolysaccharide. Arterioscler Thromb Vasc Biol.

[51] Cohen I, Rider P, Carmi Y, Braiman A, Dotan S, White MR (2010). Differential release of chromatin-bound IL-1alpha discriminates between necrotic and apoptotic cell death by the ability to induce sterile inflammation. Proc Natl Acad Sci U S A.

[52] Di Paolo NC, Shayakhmetov DM (2016). Interleukin 1α and the inflammatory process. Nat Immunol.

[53] De Buck M, Gouwy M, Wang JM, Van Snick J, Opdenakker G, Struyf S (2016). Structure and expression of different serum amyloid A (SAA) variants and their concentration-dependent functions during host insults. Curr Med Chem.

[54] De Buck M, Gouwy M, Wang JM, Van Snick J, Proost P, Struyf S (2016). The cytokine-serum amyloid A-chemokine network. Cytokine Growth Factor Rev.

[55] Huang XF, Chi W, Lin D, Dai ML, Wang YL, Yang YM (2018). Association of IL33 and IL1RAP polymorphisms with acute anterior uveitis. Curr Mol Med.

[56] Schmitz J, Owyang A, Oldham E, Song Y, Murphy E, McClanahan TK (2005). IL-33, an interleukin-1-like cytokine that signals via the IL-1 receptor-related protein ST2 and induces T helper type 2-associated cytokines. Immunity.

[57] Butchi N, Kapil P, Puntambekar S, Stohlman SA, Hinton DR, Bergmann CC (2015). Myd88 initiates early innate immune responses and promotes CD4 T cells during coronavirus encephalomyelitis. J Virol.

[58] Levine B, Kroemer G (2019). Biological functions of autophagy genes: a disease perspective. Cell.

[59] Puleston DJ, Simon AK (2014). Autophagy in the immune system. Immunology.

[60] Dinarello CA (2011). Interleukin-1 in the pathogenesis and treatment of inflammatory diseases. Blood.

[61] Mukherjee T, Hovingh ES, Foerster EG, Abdel-Nour M, Philpott DJ, Girardin SE (2019). NOD1 and NOD2 in inflammation, immunity and disease. Arch Biochem Biophys.

[62] Zhang M, Lee AJ, Wu X, Sun SC (2011). Regulation of antiviral innate immunity by deubiquitinase CYLD. Cell Mol Immunol.

[63] Oliveira AC, Gomes-Neto JF, Barbosa CD, Granato A, Reis BS, Santos BM (2017). Crucial role for T cell-intrinsic IL-18R-MyD88 signaling in cognate immune response to intracellular parasite infection. Elife.

[64] Sugiyama K, Muroi M, Kinoshita M, Hamada O, Minai Y, Sugita-Konishi Y (2016). NF-κB activation via MyD88-dependent Toll-like receptor signaling is inhibited by trichothecene mycotoxin deoxynivalenol. J Toxicol Sci.

[65] Rocha DM, Caldas AP, Oliveira LL, Bressan J, Hermsdorff HH (2016). Saturated fatty acids trigger TLR4-mediated inflammatory response. Atherosclerosis.

[66] Arnett HA, Escobar SS, Gonzalez-Suarez E, Budelsky AL, Steffen LA, Boiani N (2007). BTNL2, a butyrophilin/B7-like molecule, is a negative costimulatory molecule modulated in intestinal inflammation. J Immunol.

[67] Elenkov IJ, Iezzoni DG, Daly A, Harris AG, Chrousos GP (2005). Cytokine dysregulation, inflammation and well-being. NeuroImmunoModulation.

[68] Yang B, Wang J, Wang Y, Zhou H, Wu X, Tian Z (2013). Novel function of Trim44 promotes an antiviral response by stabilizing VISA. J Immunol.

[69] Wang GX, Cho KW, Uhm M, Hu CR, Li S, Cozacov Z (2014). Otopetrin 1 protects mice from obesity-associated metabolic dysfunction through attenuating adipose tissue inflammation. Diabetes.

[70] Ren T, Xu L, Jiao S, Wang Y, Cai Y, Liang Y (2009). TLR9 signaling promotes tumor progression of human lung cancer cell in vivo. Pathol Oncol Res.

[71] Xu L, Wang C, Wen Z, Zhou Y, Liu Z, Liang Y (2010). CpG oligodeoxynucleotides enhance the efficacy of adoptive cell transfer using tumor infiltrating lymphocytes by modifying the Th1 polarization and local infiltration of Th17 cells. Clin Dev Immunol.

[72] Liang X, Xu Z, Yuan M, Zhang Y, Zhao B, Wang J (2016). MicroRNA-16 suppresses the activation of inflammatory macrophages in atherosclerosis by targeting PDCD4. Int J Mol Med.

[73] Wang X, Zhang L, Wei Z, Zhang X, Gao Q, Ma Y (2013). The inhibitory action of PDCD4 in lipopolysaccharide/D-galactosamine-induced acute liver injury published correction appears in lab invest. Lab Invest.

[74] Wang L, Zhao M, Guo C, Wang G, Zhu F, Wang J (2016). PDCD4 deficiency aggravated colitis and colitis-associated colorectal cancer via promoting IL-6/STAT3 pathway in Mice. Inflamm Bowel Dis.

[75] Aksentijevich I, Masters SL, Ferguson PJ, Dancey P, Frenkel J, van Royen-Kerkhoff A (2009). An autoinflammatory disease with deficiency of the interleukin-1-receptor antagonist. N Engl J Med.

[76] Dinarello CA (2009). Immunological and inflammatory functions of the interleukin-1 family. Annu Rev Immunol.

