# Supplementary figures and images for: Modulation of long noncoding RNA (lncRNA) and messenger RNA (mRNA) expression in the liver of Beagle dogs by Toxocara canis infection
Source: Parasit Vectors. 2023 Mar 29;16:114. doi: 10.1186/s13071-023-05738-9 (PMC10057693; doi:10.1186/s13071-023-05738-9)

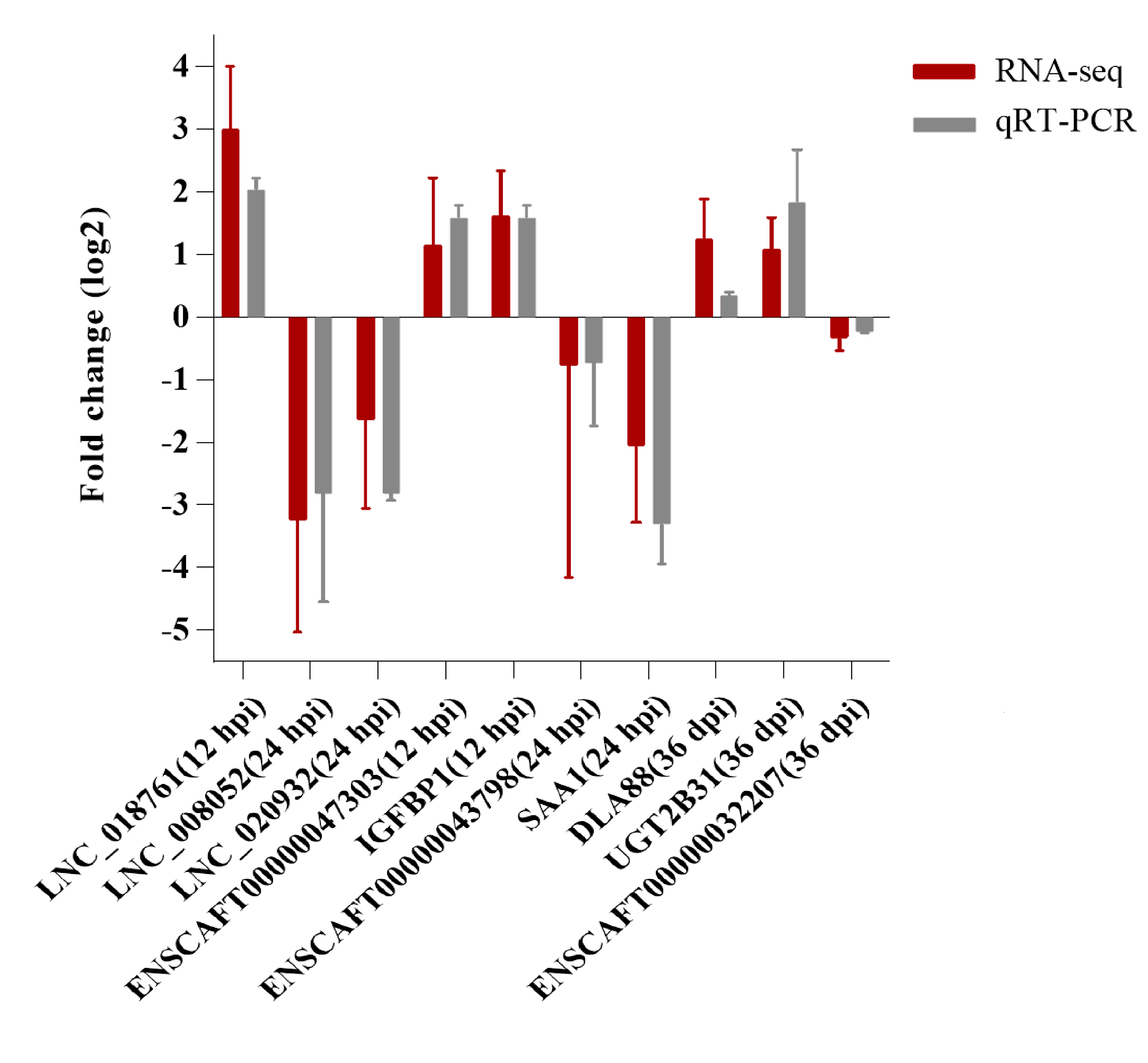

Supplement: Supplementary file 2 — Additional file 2: Figure S1. The qRT-PCR confirmation of the differentially expressed lncRNAs and mRNAs identified by RNA-Seq. The red and gray columns show the fold change values obtained by RNA sequencing and qRT-PCR, respectively. Y-axis shows the relative change of lncRNA and mRNA levels expressed as fold increase compared with the control L13A. The X-axis shows the name of the 10 RNAs used in the analysis. [file 13071_2023_5738_MOESM2_ESM.tif]

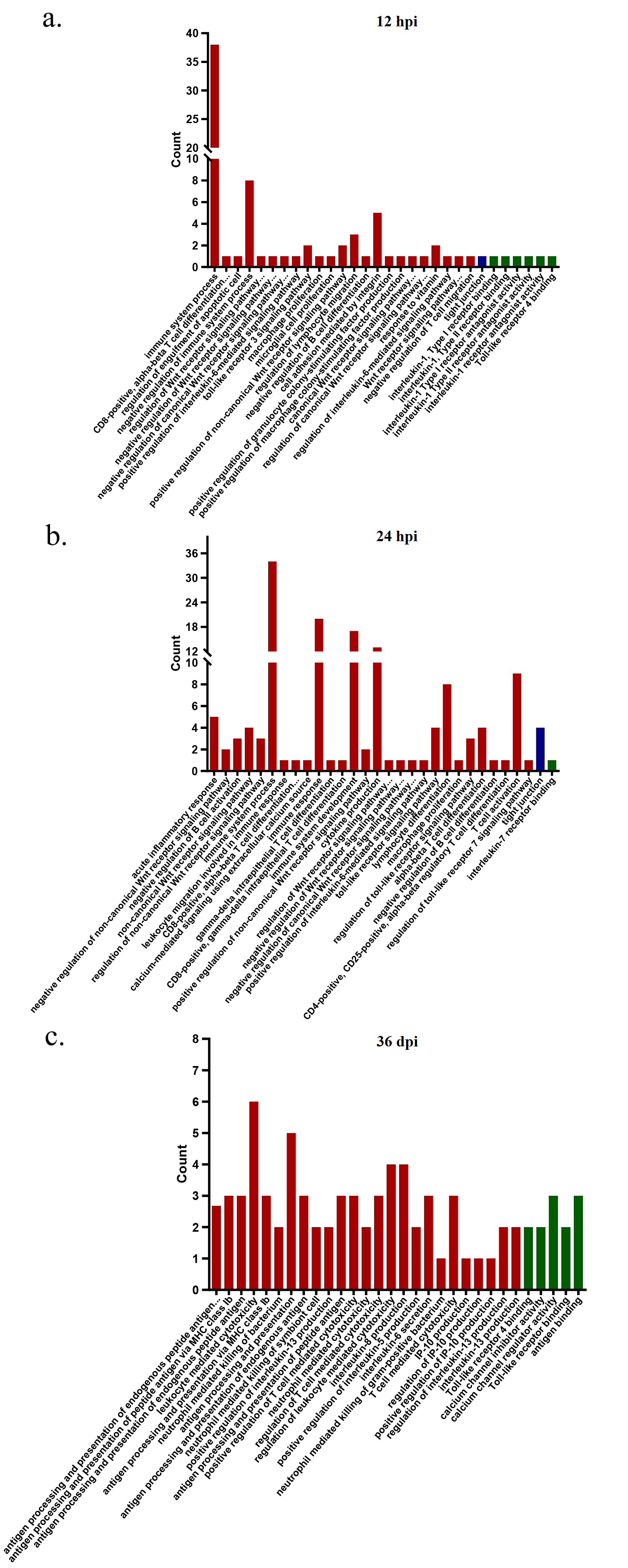

Supplement: Supplementary file 5 — Additional file 5: Figure S2. The top 30 immune- or inflammation-related GO terms of the differentially expressed (DE) mRNAs. The significantly enriched GO terms in biological process (red column), cellular component (blue column) and molecular function (green column) at (a) 12 hpi, 24 hpi (b) and 36 dpi (c). The left Y-axis shows the number of DEmRNAs. The X-axis shows the name of the GO terms. [file 13071_2023_5738_MOESM5_ESM.tif]

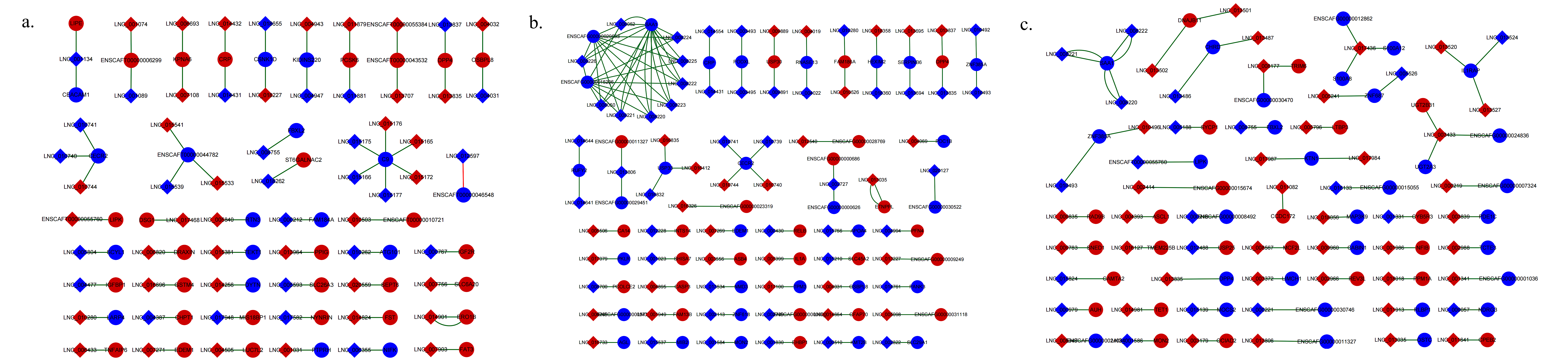

Supplement: Supplementary file 8 — Additional file 8: Figure S3. The co-localization networks of DElncRNAs and DEmRNAs at (a) 12 hpi, (b) 24 hpi and (c) 36 dpi, respectively. The diamonds represent the DElncRNAs and circles represent the DEmRNAs. The up- and down-regulated RNAs are indicated by red and blue colors, respectively. [file 13071_2023_5738_MOESM8_ESM.tif]

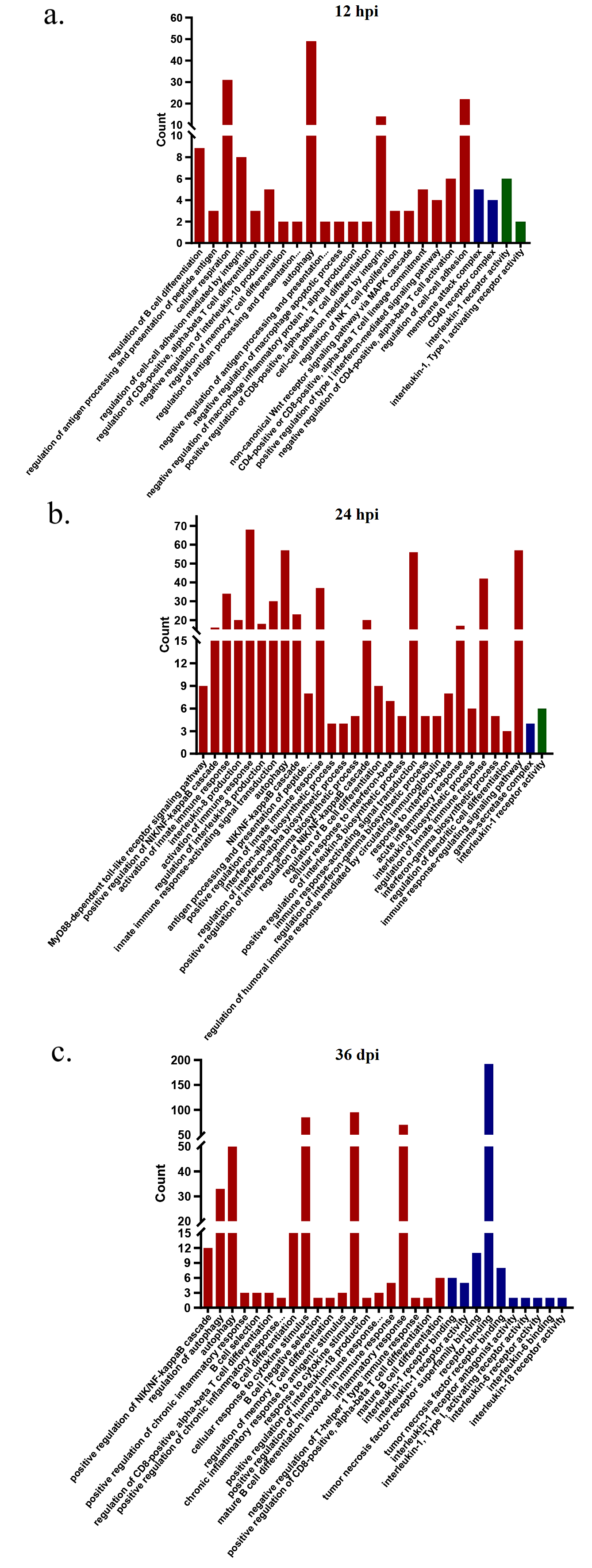

Supplement: Supplementary file 10 — Additional file 10: Figure S4. The top 30 immune- or inflammation-related GO terms of the differentially expressed (DE) lncRNAs. The significantly enriched GO terms in biological process (red column), cellular component (blue column) and molecular function (green column) terms at 12 hpi (a), 24 hpi (b) and 36 dpi (c). The left Y-axis shows the number of DElncRNAs. The X-axis shows the name of the GO terms. [file 13071_2023_5738_MOESM10_ESM.tif]
